# Personality Traits and Insomnia Symptoms in Shift Workers

**DOI:** 10.3389/fpsyg.2021.689741

**Published:** 2021-08-31

**Authors:** Brigitte Holzinger, Lucille Mayer, Gerhard Klösch

**Affiliations:** ^1^Institute for Consciousness and Dream Research, Vienna, Austria; ^2^Sleep Coaching Programme, Medical University, Vienna, Austria; ^3^Department of Neurology, Medical University of Vienna, Vienna, Austria

**Keywords:** shift work, shift irregularity, sleep quality, insomnia, personality, Big Five

## Abstract

The discrepancy between natural sleep-wake rhythm and actual sleep times in shift workers can cause sleep loss and negative daytime consequences. Irregular shift schedules do not follow a fixed structure and change frequently, which makes them particularly harmful and makes affected individuals more susceptible to insomnia. The present study compares insomnia symptoms of non-shift workers, regular shift workers, and irregular shift workers and takes into account the moderating role of the Big Five personality traits and levels of perfectionism. Employees of an Austrian railway company completed an online survey assessing shift schedules, sleep quality and duration, daytime sleepiness, and personality traits. A total of 305 participants, of whom 111 were non-shift workers, 60 regular shift workers, and 134 irregular shift workers, made up the final sample. Irregular shift workers achieved significantly worse scores than one or both of the other groups in time in bed, total sleep time, sleep efficiency, sleep duration, sleep quality, sleep latency, and the number of awakenings. However, the values of the irregular shifts workers are still in the average range and do not indicate clinical insomnia. Participants working regular shifts reported the best sleep quality and longest sleep duration and showed the least nocturnal awakenings, possibly due to higher conscientiousness- and lower neuroticism scores in this group. Agreeableness increased the effect of work schedule on total sleep time while decreasing its effect on the amount of sleep medication taken. Perfectionism increased the effect of work schedule on time in bed and total sleep time. Generalization of results is limited due to the high percentage of males in the sample and using self-report measures only.

## Introduction

Shift work, or work performed during non-traditional working hours, leads to a discrepancy between natural sleep-wake rhythm and actual sleep times. This disruption of the circadian rhythm is likely to cause insomnia and excessive daytime sleepiness (Harrington, 2001; Caruso, 2013; d’Ettorre et al., 2020; Khan et al., 2020; Wen et al., 2020), the main symptoms of shift work sleep disorder (American Academy of Sleep Medicine, 2014). The International Classification of Sleep Disorders defines insomnia as a combination of sleep initiation or maintenance problems and negative daytime consequences (American Academy of Sleep Medicine, 2014), such as an increased risk for accidents, high stress levels, difficulties in emotion regulation and social functioning, and gastrointestinal, cardiovascular, and metabolic problems (Ma et al., 2015; Angerer et al., 2017; d’Ettorre et al., 2020). Insomnia is one of the main reasons for depressive and anxiety symptoms in shift workers (Khan et al., 2020; Vigoureux and Lee, 2021).

While all shift work is challenging for our health, irregular shift schedules are particularly harmful and have been associated with higher prevalence of insomnia, sleep deprivation, and daytime sleepiness as well as a higher risk for gastrointestinal, reproductive, metabolic, and cardiovascular disorders (Härmä et al., 2002; Zverev and Misiri, 2009; Cheng and Cheng, 2017; Khan et al., 2020). Irregular shifts also increase the risk of developing burnout symptoms and job dissatisfaction (Bagheri Hosseinabadi et al., 2019). Rapidly changing shifts do not allow for an adjustment in the circadian clock, and as a result, hormonal secretion is not in line with the actual sleep-wake cycle (Lim et al., 2020). High shift intensity and short recovery periods increase the risk for insomnia (Chung et al., 2021). Closely related to sleep quality but hardly examined so far are dream characteristics of shift workers. Due to chronically heightened stress levels, shift workers, especially irregular shift workers, may tend to experience nightmares more frequently (Garcia et al., 2021). Because arousal levels tend to be higher due to stress and irregular sleep schedules, there are more frequent nighttime awakenings, possibly leading to increased dream recall (Schredl and Reinhard, 2008).

While it is a common belief that people react differently to the demands of shift work, and despite the large number of studies on the subject of shift work and sleep, there are hardly any studies that examine the role of personality traits in this context. The five-factor model of personality traits (the Big Five) has emerged in the last decades of the past century (McCrae and Costa, 1989, 1999) and has become the most widely accepted framework when studying personality factors (Greenlees, 2020). The model postulates that all “personality traits can be classified under five subordinate dimensions” (Greenlees, 2020), namely, *openness to experience*, *conscientiousness*, *extraversion*, *agreeableness*, and *neuroticism* (McCrae and Costa, 2008). Neuroticism has been found to be a significant negative predictor for shift work tolerance (Ebrahimi and Pordanjani, 2013; Foldal et al., 2016) and has been suggested to predispose workers to develop burnout syndrome, while agreeableness and openness seem to protect against burnout (De la Fuente-Solana et al., 2021). Shift workers with higher levels of neuroticism also report poorer sleep quality (Booker et al., 2018) and higher levels of insomnia (Larsgård and Saksvik-Lehouillier, 2017). On the other hand, extraversion is associated with better sleep quality (Stephan et al., 2018) and higher levels of extraversion in shift workers are associated with less symptoms of insomnia (Larsgård and Saksvik-Lehouillier, 2017). Agreeableness and conscientiousness are typically associated with better sleep quality, whereas openness is usually not associated with sleep (Hintsanen et al., 2014); however, findings on these three personality traits vary more across studies (Stephan et al., 2018). *Perfectionism* is another important personality that entails “unrealistically high and rigid standards for performance, fear of failure, excessive self-criticism, and the inability to derive satisfaction from their achievements” (Azevedo et al., 2010, p. 476). It has been suggested that high levels of perfectionism are dysfunctional and associated with psychopathology (Pacht, 1984). Accordingly, studies have reported that perfectionism scores are a predictor of sleep disturbances (Azevedo et al., 2010) and that being a perfectionist increases the vulnerability to work-related stressors and the risk of developing burnout (Hewitt and Flett, 1993).

The aim of this study was to look more closely at insomnia symptoms reported by shift workers employed by the Austrian Railway Company *Österreichische Bundesbahnen*. To our knowledge, this is the first study to compare sleep-related aspects of non-shift workers, regular shifts workers, and irregular shift workers. It will also be investigated whether there are differences in dream recall and nighttime awakenings due to nightmares. Beyond that, this study assessed the moderating role of the Big Five personality traits and perfectionism scores on the relationship between shift schedule and insomnia symptoms.

## Materials and Methods

### Study Design and Participants

Two questionnaires were made available online^1^ between June 2017 and April 2020 as part of a sleep coaching program especially designed for shift workers. Participants were informed about the objectives and methods of this survey, and all subjects had to give informed consent before starting the online survey. All responses were analyzed and condensed into feedback reports distributed to the participants during seminars organized by the Institute for Consciousness and Dream Research in Vienna. Of the 375 participants who completed the survey, 70 were excluded due to missing demographic data. This led to a final sample of 305 (258/84.6% males; aged between 20 and 64, *M*_age_ = 46.40, *SD*_age_ = 8.89). Of these 305 participants, 111 (80/72.1% males) worked in non-shift occupations and 194 (178/91.8% males) worked in shifts, with 60 rating their shift schedule as regular and 134 as irregular.

### Measurements

Participants filled in one self-rated questionnaire, including 40 items assessing demographic data regarding age, sex, weight and height, shift schedule, sleep quantity and quality, and daytime sleepiness. In addition, all participants were offered the possibility to fill in a 14-day sleep diary containing more detailed questions about sleep habits and dreams. Only items relevant for this study will be listed in the following.

#### Shift Schedules

Participants were asked to characterize their shift plan in more detail. It was assessed whether they worked nights or weekends, whether they worked in 2-shift or 3-shift systems or other, and whether they suffered from any complaints due to shift work. Participants then rated whether they worked in regular (constant over the course of multiple weeks) or irregular shift schedules. According to the company’s shift schedules we received beforehand, irregular shifts imply mostly unpredictable schedules that change from week to week and may even be on-demand schedules, while regular shifts are constant over longer periods of time, often years, and typically contain two 12-h night shifts per week with long recovery periods in between.

#### Sleep Habits

Participants completed the Pittsburgh Sleep Quality Index (PSQI; Buysse et al., 1989) validated German version (Backhaus et al., 2002), an established measurement for sleep quality. Based on seven components, namely, *subjective sleep quality*, *sleep latency*, *sleep duration*, *habitual sleep efficiency*, *sleep disturbances*, use of *sleeping medication*, and *daytime dysfunction*, a total score between 0 and 21 was calculated. A score of 21 indicated the poorest sleep quality, and 0, no sleep problems at all. A total score >5 indicates poor sleep quality (Backhaus et al., 2002). For each subscale, partial scales were calculated (ranging from 0 to 3). In case of sleep latency, two item scores were summed and transformed into a new score ranging from 0 to 3. Additionally, one item of the PSQI questionnaire was analyzed separately: “How often in the last 4 weeks did you suffer from sleep disorders, because you experienced bad dreams?” Possible answers were “Not at all” = 1, “less than once a week” = 2, “once or twice a week” = 3, and “3 times or more per week” = 4.

The 14-day sleep diary included items that allowed the calculation of average scores for *time in bed* in minutes, *total sleep time* in minutes, and *sleep efficiency* as the proportion of total sleep time to time in bed. One item assessed the total *number of awakenings* per night.

#### Daytime Sleepiness

The Epworth Sleepiness Scale (Johns, 1991) validated German version (Bloch et al., 1999) was included to measure *daytime sleepiness*. Participants rated the likelihood to doze off or fall asleep in eight everyday situations (e.g., watching TV) on a scale from 0 (“would never doze off”) to 3 (“high chance to doze off”) to obtain a total score between 0 and 24 (where 24 indicated the highest diurnal fatigue). Scores >10 indicate critical daytime sleepiness (Johns, 1992).

#### Personality Traits

Participants rated their perfectionism and burnout levels by two questions about perfectionism (“Do others think you are a perfectionist?” and “Do you think you are a perfectionist?”) and two questions addressing burnout-risk (“Do you often feel lack of energy?” and “Do you sometimes feel confused, as if not quite yourself?”). Responses to these six items ranged on a 4-point scale from “not at all true” (0 points), “partly true” (1 point) to “mostly true” (2 points), and “completely true” (3 points). An average score was then calculated for the two categories burnout and perfectionism.

The BFI-10 (Rammstedt et al., 2017) was used for the assessment of the Big Five personality traits “openness,” “conscientiousness,” “extraversion,” “agreeableness,” and “neuroticism.” It contains ten items, two for each trait, and each participant was assigned a score of 0 (below average), 1 (average), or 2 (above average).

#### Burnout Risk

Two questions asked about the current burnout risk (“Do you often feel lack of energy?” and “Do you sometimes feel confused, as if not quite yourself?”). Responses to these two items ranged on a 4-point scale from “not at all true” (0 points), “partly true” (1 point) to “mostly true” (2 points), and “completely true” (3 points). An average score was then calculated.

### Statistical Analysis

The three groups non-shift, regular shift, and irregular shift workers were statistically compared. Means and standard deviations were calculated for each variable and each group. For PSQI items that are ordinally scaled, median and interquartile range were calculated. These values were then examined for significant differences: ANOVA was used to check for significant differences between groups, and contrasts were calculated for all but the PSQI items, Kruskal-Wallis tests were used to check for differences regarding the PSQI items with subsequent implementation of the Dunn-Bonferroni test. Multiple regression analyses were conducted to assess the role of the Big Five personality traits and perfectionism. The stepwise method was used to first include the Big Five personality traits and perfectionism in the model and see whether they were able to predict the occurrence of insomnia symptoms. In the next step, personality characteristics were also included as moderators or interaction terms (shift group × personality trait). The data were tested beforehand and met all assumptions with the exception for linear relationship. For those insomnia symptoms showing a non-linear relationship, quadratic terms were included in the model (shift group and shift group squared).

The threshold for the rejection of the null hypothesis was set to 0.05 and adapted when calculating multiple regressions. All statistical analyses were performed using the IBM SPSS Statistics for Windows, version 26.0 (Armonk, NY, United States).

## Results

For a comparison of the three groups, non-shift, regular shift and irregular shift workers, means and standard deviations of personal characteristics age, perfectionism, and Big Five personality characteristics were calculated and are presented in Table 1. Differences were found regarding perfectionism, conscientiousness, and neuroticism. Table 2 shows the results of the ANOVA contrasting the three groups. All significant results are marked by bold letters.

**Table 1:** Means and standard deviations of age, perfectionism, and Big Five personality traits of each group.

	Non-shift		Regular shift		Irregular shift	
	*M*	*SD* ^*^	*M*	*SD*	*M*	*SD*
Age	46.22	10.15	47.08	8.70	46.04	7.98
Perfectionism	1.77	0.70	1.61	0.76	1.51	0.71
Openness	0.96	0.51	1.05	0.54	0.97	0.55
Conscientiousness	0.79	0.50	1.03	0.57	0.98	0.52
Extraversion	1.21	0.69	0.98	0.57	0.98	0.60
Agreeableness	0.89	0.42	0.75	0.48	0.79	0.50
Neuroticism	1.32	0.55	0.97	0.69	1.18	0.63

* SD, standard deviation.

**Table 2:** ANOVA contrast tests between non-shift workers, regular shift workers, and irregular shift workers.

Characteristics	Value of Contrast		
	Regular shift work–non-shift work	Irregular shift work–non-shift work	Irregular shift work–regular shift work
Age	0.87	−0.17	−1.04
Perfectionism	−0.16	**−0.25** ^**^	−0.10
Openness	0.09	0.00	−0.08
Conscientiousness	**0.25** ^*^	0.20	−0.05
Extraversion	−0.23	−0.23	0.00
Agreeableness	−0.15	−0.10	0.04
Neuroticism	**−0.36** ^*^	−0.14	**0.21** ^*^

All significant values are presented in bold letters.

* *p* < 0.05;

** *p* < 0.01.

Conscientiousness was significantly higher in workers with regular shift work than non-shift workers. Neuroticism was lower in regular shift workers as compared to non-shift workers and higher in irregular than in regular shift workers. Perfectionism was lower in irregular shift workers, as compared to non-shift workers.

Tables 3 and 4 show how the three groups differed regarding insomnia symptoms. Table 3 presents means and standard deviations, respectively, median and interquartile range for ordinally scaled variables, while pairwise comparisons are shown in Table 4. Those participants working in regular shifts reported higher sleep efficiency, higher total sleep time, best sleep duration (PSQI), least nocturnal awakenings, higher sleep quality (PSQI global score), lower sleep onset latencies (in minutes), and least sleep disorders due to nightmares, and took the least sleep medication. Daytime sleepiness was highest in regular shift workers.

**Table 3:** Means and standard deviations, respectively, medians and interquartile ranges of insomnia-related symptoms for each group.

	Non-shift		Regular shift		Irregular shift	
	*M*	*SD*	*M*	*SD*	*M*	*SD*
Sleep efficiency (percent, diary)	86.93	9.59	88.19	11.39	86.54	10.22
Sleep efficiency (PSQI)	0.45	0.87	0.45	0.79	0.72	1.06
Time in bed (minutes, diary)	454.80	64.20	438.60	69.60	426.00	62.40
Total sleep time (minutes, diary)	393.00	60.60	401.40	81.00	367.80	70.20
Number of awakenings per night (diary)	1.54	1.21	1.04	0.77	1.68	1.55
Sleep quality (PSQI global score)	6.60	2.87	5.77	2.45	7.14	2.89
Sleep onset latency (minutes, diary)	26.15	23.14	25.28	20.14	29.50	26.23
Daytime sleepiness (ESS)	8.25	4.22	8.35	3.80	7.57	3.90
Burnout risk	1.29	0.59	1.26	0.54	1.43	0.56
Dream recall frequency (diary)	0.79	0.77	1.04	0.87	1.25	0.85
Sleep disorders due to nightmares (item PSQI)	1.69	0.85	1.41	0.76	1.43	0.80
	**Median**	**IQR**	**Median**	**IQR**	**Median**	**IQR**
Sleep duration (PSQI)	0	1	0	1	0	1
Sleep onset latency (PSQI)	1	1	1	1	2	1
Sleep medication (PSQI)	0	0	0	0	0	0

**Table 4:** Pairwise comparisons between non-shift workers, regular shift workers, and irregular shift workers (ANOVA contrast tests for metrically scaled variables, respectively, Kruskal-Wallis tests and Dunn-Bonferroni tests for ordinally scaled variables).

	Value of Contrast (ANOVA)		
	Regular shift work–non-shift work	Irregular shift work–non-shift work	Irregular shift work–regular shift work
Sleep efficiency (diary)	1.26	−0.40	−1.66
Sleep efficiency (PSQI)	0.00	**0.27** ^*^	**0.27** ^*^
Time in bed (minutes, diary)	−0.27	**−0.48** ^**^	−0.21
Total sleep time (minutes, diary)	0.14	**−0.43** ^*^	**−0.57** ^*^
Number of awakenings per night (diary)	**−0.50** ^*^	0.13	**0.64** ^*^
Sleep quality (PSQI global score)	−0.84	0.54	**1.38** ^*^
Sleep onset latency (minutes, diary)	−0.84	3.35	4.22
Daytime sleepiness (ESS)	0.10	−0.69	−0.78
Burnout risk	−0.03	0.14	0.17
Dream recall frequency (diary)	0.25	**0.46** ^*^	0.21
Sleep disorders due to nightmares (item PSQI)	−0.28	**−0.25** ^*^	0.03
	**Pairwise Comparisons (Dunn-Bonferroni Tests)**		
Sleep duration (PSQI)	**2.83** ^*^	0.17	**2.78** ^*^
Sleep onset latency (PSQI)	Kruskal-Wallis test, non-significant (Chi-square = 4.27, *p* = 0.12, *df* = 2)		
Sleep medication (PSQI)	Kruskal-Wallis test, non-significant (Chi-square = 2.36, *p* = 0.31, *df* = 2)		

All significant values are presented in bold letters.

* *p* < 0.05;

** *p* < 0.01.

Contrasts are presented to allow for a more detailed evaluation of the differences between the three shift groups and see which differences were statistically significant. Sleep efficiency (calculated from the PSQI) was significantly lower in irregular shift workers as compared to non-shift workers and regular shift workers. Time in bed was lower in irregular shift workers than non-shift workers. Total sleep time was lower in irregular shift workers as compared to both, non-shift workers and regular shift workers. Sleep duration (calculated from the PSQI) was higher in workers on regular shifts as compared to non-shift workers but lower in irregular shift workers than the other two groups (best sleep duration in regular shifts). The number of awakenings was lower in regular shifts than non-shifts and higher in irregular shifts than regular shifts (lowest number of awakenings in regular shifts). Sleep quality (PSQI global score) was lower in irregular shifts than regular shifts. Sleep onset latency was higher in irregular shifts than non-shift. Differences in the consumption of sleep medication were non-significant. Dream recall was better in irregular shift workers than non-shift workers. Irregular shift workers reported less sleep problems due to nightmares than non-shift workers.

**Figure 1:**
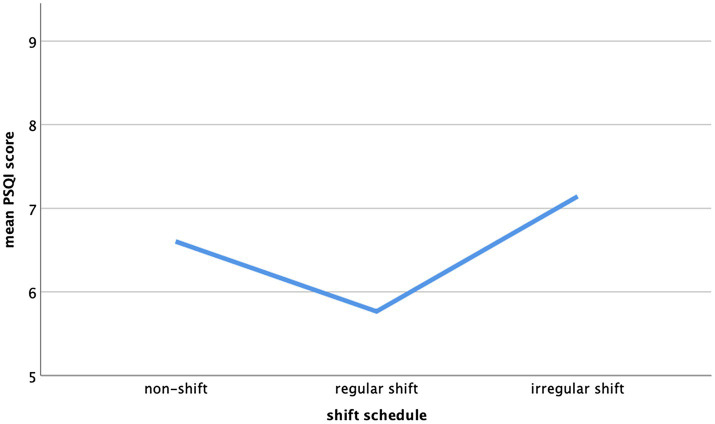
Mean Pittsburgh Sleep Quality Index (PSQI) score of non-shift workers, regular shift workers, and irregular shift workers.

**Figure 2:**
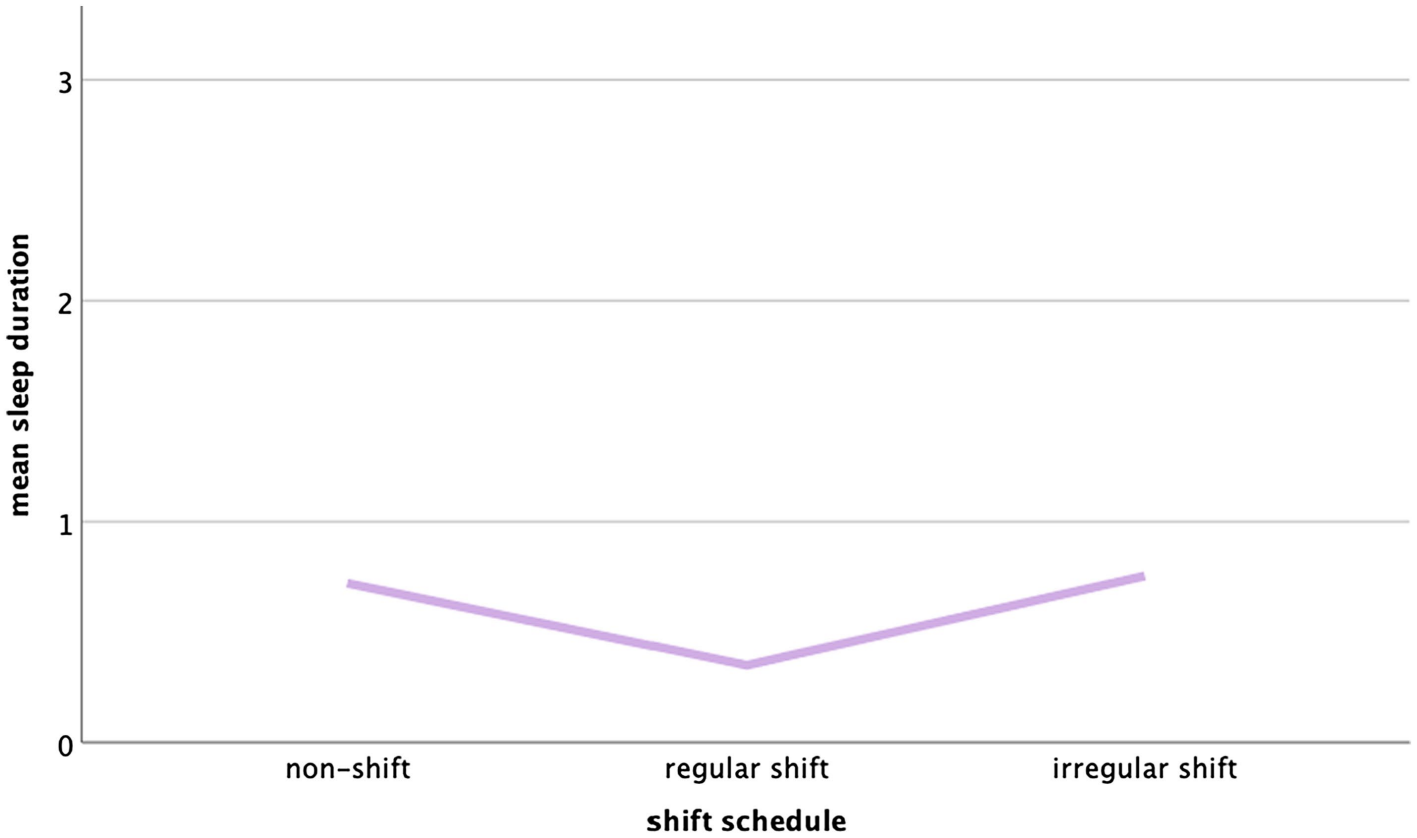
Mean sleep duration (PSQI) in non-shift workers, regular shift workers, and irregular shift workers.

The relationship between type of shift schedule and sleep duration as well as shift schedule and sleep quality was non-linear but u-shaped (see Figures 1, 2).

For these variables, group (non-shift, regular shift, and irregular shift) and its quadratic term group^2^ were included in the regression model. Overall, the relationship between shift schedule and sleep efficiency (PSQI), time in bed, and total sleep time was linear and significant, the relationship between shift schedule and sleep duration and sleep quality was non-linear and significant, and the relationship between shift schedule and sleep efficiency, number of awakenings, sleep latency, daytime sleepiness, sleep medication, and burnout risk did not reach significance.

Depending on the number of variables, the sample size varied between *N* = 100 and *N* = 211. Using the stepwise method, it was found that the model explained a significant amount of the variance in time in bed [*F*(8, 100) = 2.86, *p* = 0.007, *R*^2^ = 0.19, *R*^2^Adjusted = 0.12], sleep quality PSQI [*F*(8, 204) = 2.42, *p* = 0.016, *R*^2^ = 0.09, *R*^2^Adjusted = 0.05], sleep latency PSQI [*F*(7, 205) = 2.14, *p* = 0.041, *R*^2^ = 0.07, *R*^2^Adjusted = 0.04], and sleep medication PSQI [*F*(9, 203) = 3.38, *p* = 0.001, *R*^2^ = 0.13, *R*^2^Adjusted = 0.09], but not in sleep efficiency [*F*(6, 103) = 1.18, *p* = 0.322, *R*^2^ = 0.06, *R*^2^Adjusted = 0.01], sleep efficiency PSQI [*F*(6, 206) = 0.98, *p* = 0.44, *R*^2^ = 0.03, *R*^2^Adjusted = 0.00], total sleep time [*F*(8, 100) = 3.96, *p* = 0.004, *R*^2^ = 0.20, *R*^2^Adjusted = 0.13], sleep duration PSQI [*F*(7, 205) = 1.66, *p* = 0.120, *R*^2^ = 0.05, *R*^2^Adjusted = 0.02], number of awakenings [*F*(7, 100) = 1.58, *p* = 0.151, *R*^2^ = 0.10, *R*^2^Adjusted = 0.04], sleep latency [*F*(7, 201) = 1.21, *p* = 0.30, *R*^2^ = 0.04, *R*^2^Adjusted = 0.01], awakenings due to nightmares [*F*(6, 152) = 0.20, *p* = 0.978, *R*^2^ = 0.01, *R*^2^Adjusted = −0.03], or dream recall frequency [*F*(8, 99) = 2.02, *p* = 0.052, *R*^2^ = 0.14, *R*^2^Adjusted = 0.07].

The level of agreeableness was a significant predictor for time in bed [*B* = 0.69, *p* = 0.007] and sleep medication [*B* = 0.36, *p* = 0.001]. Agreeableness was a significant moderator on the effect of the type of work schedule on total sleep time [*B* = 0.87, *p* = 0.004] and sleep medication [*B* = −0.39, *p* = 0.001]. The higher the level of agreeableness, the bigger the effect of work schedule on total sleep time, and the smaller the effect of work schedule on sleep medication. Perfectionism was a significant moderator on the effect of work schedule on time in bed [*B* = 0.55, *p* = 0.005] and total sleep time [*B* = 0.60, *p* = 0.005]. The higher the level of perfectionism, the bigger the effect of work schedule on total sleep time and time in bed.

## Discussion

Overall, shift workers who rated their shift schedules as irregular achieved worst scores in all insomnia-related variables compared to non-shift workers and regular shift workers. Irregular shift workers differed significantly and negatively from one or both of the other groups in time in bed, total sleep time, sleep efficiency (PSQI), sleep duration (PSQI), sleep quality (PSQI global score), sleep latency (PSQI), and the number of awakenings. In other words, irregular shift workers slept less, worse, needed longer to fall asleep, and woke up more often during the night. This is in line with former findings indicating that sleep complaints are more common in irregular shifts (Härmä et al., 2002), and have been suggested to be even more common than in case of permanent night shifts (Pilcher et al., 2000). Even if these results indicate a decrease in sleep quality with increasing shift irregularity, it is not possible to speak of clinical insomnia in this case, since the values of the irregular shift workers are nevertheless in average range, with sleep efficiency >86%, sleep latency <30 min, nocturnal awakenings <2, total sleep time >360 min, and daytime sleepiness <9. Only the global PSQI score is >5 and therefore clinically relevant (Backhaus et al., 2002). However, most participants reported quite serious sleep and daytime issues during the seminars and the fact that no clinical insomnia scores were obtained does not detract from the relevance of the fact that irregular shift workers sleep significantly worse than the other two groups studied. It should be mentioned here again that the results of the diary and the PSQI do not always match, which could have different reasons that cannot be clarified here with certainty. Presumably, the items of the PSQI can be considered more reliable, since they have been validated in numerous languages and versions. However, the advantage of the diary is that it collects data over a longer period of time; for example, time in bed and actual sleeping hours are reported per day over a period of 2 weeks to allow for a calculation of a more representative average value.

No differences were found regarding daytime sleepiness, which is surprising since the quality of sleep and the fatigue experienced during the day are typically closely related (Cotrim et al., 2017).

Furthermore, irregular shift workers showed a higher dream recall frequency than non-shift workers and regular shift workers. Since working in shifts disturbs the circadian rhythms and certain environmental conditions, such as lighting, make it more difficult to fall asleep, nocturnal awakenings occur more often, and a higher proportion of sleep is spent in rapid eye movement (REM) sleep – which in turn increases the likelihood of experiencing dreams. However, very few studies have assessed dream characteristics of shift workers. Only one study compared dream recall in shift workers and non-shift workers and found no differences (Stepansky et al., 1998). In line with the finding that irregular shift workers sleep worse than regular shift workers, it seems plausible that they spent even more time in REM sleep and therefore experience and remember more dreams.

Interestingly, irregular shift workers reported less sleep disturbances due to nightmares compared to non-shift workers and regular shift workers.

Surprisingly, in this sample, those participants working on regular shifts scored best in sleep quality, had a longer sleep duration, and reported the least nocturnal awakenings; thus, they achieved better results than non-shift workers. Almost all remaining variables showed the same tendency without reaching significance. Overall, a u-shaped relationship was found with regular shift workers showing the least insomnia symptoms.

When looking at personality traits, the three groups differed significantly in neuroticism, conscientiousness, and perfectionism, which may be an explanation for why regular shift workers reported less sleep issues than non-shift workers.

Individuals with high neuroticism scores show greater emotional instability than those with lower scores and are believed to have greater activation of the limbic system when responding to environmental stressors (Eysenck, 1967; Harvey et al., 2014). Therefore, they tend to associate sleep and sleeping environment with negative emotions after only one episode of sleep disruption, and neuroticism together or high arousability may lead to disrupted sleep (Harvey et al., 2014). It has been shown that neuroticism and rigidity play a role as predictors of shift work tolerance, with neurotic and rigid individuals experiencing greater difficulties than others (Ebrahimi and Pordanjani, 2013) and that people with characteristics similar to neuroticism have more difficulties adapting their circadian rhythms (Hennig et al., 1998). While elevated scores in neuroticism harbor risks, inconspicuous scores can have protective effects. In this sample, regular shift workers achieved the lowest scores in neuroticism, which may explain why this group reported the least sleep issues. Interestingly, former studies have shown that neuroticism increases with years of shift work (Harrington, 2001).

Regular shift workers also achieved significantly higher levels of conscientiousness than non-shift workers. Conscientiousness comprises the three aspects achievement orientation, dependability, and orderliness, and is related to degree of self-control, need for achievement, order, and persistence (Judge et al., 1999). Higher levels of conscientiousness may enable people to plan ahead, ensure a healthy work-life balance, and adhere to more regular daily schedules. These assumptions are supported by the earlier finding that conscientiousness is negatively associated with PSQI scores, while higher levels of neuroticism correlate with higher PSQI scores (Kim et al., 2015). This supports the idea that differences in personality may be the reason for better sleep quality in regular shift workers than non-shift workers.

Former studies reported that perfectionism scores, especially socially prescribed perfectionism, are a predictor of sleep disturbances (Azevedo et al., 2010) and that being a perfectionist increases the vulnerability to work-related stressors (Hewitt and Flett, 1993). However, perfectionism is suggested to be a multidimensional construct which combines positive and negative (maladaptive) components, having opposite effects on health (Magnusson et al., 1996). In the present study, regular shift workers reported to be less perfectionist than non-shift workers, which may be an explanation for better sleep quality scores in this group. However, irregular shifts workers reported to be the least perfectionist subgroup, which is not in line with the other results. Perhaps, participants associated perfectionism with different aspects of the construct, and this may have led to less clear results.

The Big Five trait agreeableness significantly predicted both time in bed and the amount of consumed sleep medication and increased the effect of work schedule on total sleep time while decreasing its effect on the amount of sleep medication taken. Perfectionism increased the effect of work schedule on time in bed and total sleep time.

Contrary to former findings, no differences in burnout risk were reported. For instance, one study (Jamal, 2004) reported an association between persistent non-standard work schedules and increased emotional exhaustion, stress, and burnout in Canadian workers, and another reported between shift duration and higher burnout risk among nurses (Stimpfel et al., 2012).

### Limitations

The selection of the sample may have been biased, since participation in the seminar and completion of the survey were voluntary. Another limitation is that only self-reported data were used instead of information, such as medical reports or activity monitoring by actigraphs. No formerly validated instruments were used for the assessment of burnout and perfectionism. Also, the analyzed sample consisted to a very large proportion of male participants, and therefore, generalization of results is limited. Future studies should include a multidimensional assessment of perfectionism, measuring positive and negative perfectionism separately.

## Conclusion

Irregular shift work compared to non-shift work and regular shift work was associated with significantly higher scores in almost all symptoms of insomnia. This is in line with former findings showing that irregular shift schedules, particularly those with short recovery periods in-between shifts, are most detrimental for our health. However, while shift irregularity was associated with greater sleep problems, the scores of irregular shift workers were within the average range and therefore should not be taken as evidence of a higher risk of clinically relevant insomnia. Even though research in this field has been going on for decades and shown how problematic shift work is, an ever-larger proportion of society is working in changing shifts. In order to avoid health and financial costs, it is urgently necessary to adapt working conditions and to give shift workers the opportunity to learn more about the functions of sleep. Additionally, for some of the variables, a u-shaped relationship was found, with regular shift workers rating their sleep quality as better than non-shift workers did. It could be shown that, among other things, conscientiousness and neuroticism play a decisive role in coping with stressful working conditions. Future studies should follow-up on these findings, as they suggest that some individuals are better suited to irregular shift schedules than others and personality testing may be one way to minimize negative health effects of shift work in society.

## Data Availability Statement

The datasets generated for this study can be made available upon request.

## Ethics Statement

Ethical review and approval was not required for the study on human participants in accordance with the local legislation and institutional requirements. The patients/participants provided their written informed consent to participate in this study.

## Author Contributions

GK and BH designed the research project and the tools used. BH and LM conducted the literature search and selected the eligible studies. LM drafted the manuscript. The authors confirm being the only contributors of this work and have approved it for publication.

## Conflict of Interest

The authors declare that the research was conducted in the absence of any commercial or financial relationships that could be construed as a potential conflict of interest.

## Publisher’s Note

All claims expressed in this article are solely those of the authors and do not necessarily represent those of their affiliated organizations, or those of the publisher, the editors and the reviewers. Any product that may be evaluated in this article, or claim that may be made by its manufacturer, is not guaranteed or endorsed by the publisher.

## Footnotes

^1^ www.sleepcoaching.org
